# Carpal Implants in the Current Surgical Landscape: An Illustrated Overview

**DOI:** 10.3390/jpm15120575

**Published:** 2025-11-28

**Authors:** Antonius A. van den Hurk, Lisa E. Ramaut, Rutger M. Schols, Xavier H. A. Keuter, René R. W. J. van der Hulst

**Affiliations:** 1 Department of Plastic, Reconstructive, and Hand Surgery, Maastricht University Medical Center+, 6229 Maastricht, The Netherlands; 2 Department of Plastic, Reconstructive and Aesthetic Surgery, University Hospital Brussels, Free University Brussels (VUB), 1090 Brussels, Belgium; 3 Department of Plastic, Reconstructive and Hand Surgery, Zuyderland Medical Center, 6162 Sittard-Geleen, The Netherlands

**Keywords:** implant arthroplasty, carpal arthroplasty, carpal arthritis, carpal implants

## Abstract

**Background**: Osteoarthritis of the hand and wrist is common and can have a significant impact on quality of life. Since the carpus consists of multiple bony structures, osteoarthritis can exist in many forms. Arthroplasty using implants has become the standard treatment for many larger joints. In carpal arthroplasty, many implants exist. To determine the most suitable implant in each individual patient, knowledge of the possibilities is important. This article aims to provide an overview of the more commonly used implants in carpal arthroplasty. **Methods**: This narrative review describes all types of carpal implants, namely, total wrist implants, first carpometacarpal implants, the Amandys® implant, the Pyrocardan® disc, pyrocarbon capitate resurfacing implants, the adaptive proximal scaphoid implant, and total carpal replacement. A literature search was conducted separately for each implant. All studies investigating implant performance were included. **Results**: Naturally, each implant showed different results. Implant arthroplasty is most often compared to either arthrodesis or resection arthroplasties. For joint replacing implants like total wrist implants or first carpometacarpal implants, the use of joint replacing implants seems to be beneficial in certain populations. Joint resurfacing implants show similar functional results to the comparable non-implant options. However, research on these implants is less prevalent. Regarding all implants, complication rates seemed either similar to or in favor of non-implant options. Almost no studies found a lower complication rate for implant usage. **Conclusions**: Carpal implants are a valid option in carpal osteoarthritis. Implants seem to perform comparably to non-implant options. Careful patient selection is required, especially since implants may carry a higher risk of complications.

## 1. Introduction

Joint osteoarthritis is a widespread healthcare problem. It is estimated that 13% of the global population is affected by osteoarthritis. In the population aged greater than 65 years, this number increases to 80–90% [1]. Much variance exists in the reported incidence of hand and wrist osteoarthritis. However, the most recent global burden of disease study shows an increase in hand and wrist osteoarthritis over the last decades [2,3]. Trapeziometacarpal arthritis, scaphotrapeziotrapezoid (STT) arthritis, proximal carpal row arthritis, and radioscapholunate (RSL) arthritis are some of the more common types of carpal osteoarthritis [4,5,6]. Moreover, traumatic injuries of the carpal bones may lead to complex biomechanical changes in the carpal region like scapholunate advanced collapse (SLAC) or scaphoid nonunion advanced collapse (SNAC) [7,8].

Since the cause of symptoms in osteoarthritis is the degeneration of cartilage in the joint space causing bony contact, surgical treatment is often indicated. Most often, surgical treatment of carpal arthritis involves resecting a carpal bone either fully or partly. The aim is to relieve the pain caused by bony contact. However, after resection arthroplasty, the biomechanics of the joint are altered. Implant arthroplasty also aims to relieve complaints by eliminating the painful bony contact, in this case, by restoring anatomical function of the joint using implants. Treatment of osteoarthritis using implants is already established in the current medical landscape [9]. While carpal arthroplasty is used widely in orthopedic surgeries [10], it is still emerging in the field of hand surgery [11,12].

Since the hand comprises multiple carpal bones, forming even more joint surfaces, the hand is susceptible to different forms of osteoarthritis. Therefore, a ‘one size fits all’ implant does not exist, and each patient should be carefully assessed to determine the best-fitting implant. Carpal arthroplasty involves a lot of different surgical modalities. Some are being utilized more often like arthroplasty of the trapeziometacarpal joint or total wrist arthroplasty [13]. However, some procedures are still only used selectively like total scaphoid arthroplasty or lunate arthroplasty [14,15]. Since data on very specific arthroplasty procedures may be available in limited quantities, the spectrum of the possibilities in carpal arthroplasty remains somewhat unclear. This narrative review aims to provide a comprehensive overview of the most common carpal arthroplasties based on the most recent literature.

## 2. Methods

This study is a narrative review including all types of carpal implants. To investigate the current state of research, a literature search was performed. After identification of the implants based on the literature and the authors’ experience, PubMed was searched to find all papers that describe the respective implants. The exact search prompts can be found in the Supplementary Materials. We included all studies concerning the applicable implant and primarily investigating the implant performance. Case series were included, and case reports were excluded for volume reasons. However, in the case of total scaphoid or lunate arthroplasty, we did include case reports due to a lack of other studies. For each separate search, the results were screened manually on title and abstract by the first author. The results of the search are described separately in each section. In addition, dedicated medical figures were created for each type of implant.

The implants were split into two groups, the first being joint replacing implants; these implants eliminate both the original joint surfaces after which the implant itself includes a component of articulation. The second group is joint resurfacing implants in which only one joint surface is either replaced or resurfaced.

## 3. Results

### 3.1. Joint Replacing Implants

#### 3.1.1. Total Wrist Arthroplasty

Arthroplasty in which the entire wrist joint is replaced is one of the more routinely performed procedures in orthopedic surgery. For example, in the Netherlands, according to the Dutch arthroplasty registry, 235 total wrist arthroplasties were performed between 2017 and 2023 [16]. However, this is most likely under-reported, since registration completeness in the Dutch arthroplasty registry is not complete for hand and wrist implants [17]. Total wrist arthroplasty (TWA) was introduced in 1890 with the use of ivory implants. However, it was not until the 1960s and 1970s that wrist implants gained traction alongside the rise in hip arthroplasties [18]. First-generation implants were silicone implants. The second generation involved more metal-on-polyethylene articulation, while the third generation added anatomical orientations. The fourth generation added more modularity and relied less on cement [19].

Indication

The most common indication for TWA is rheumatoid arthritis. This group of patients represents 51–71% of all patients receiving TWAs [20,21,22]. The second most common indication remains general osteoarthritis or post-traumatic osteoarthritis. This group most commonly consists of a younger, more active population [20]. Since TWA is a total arthroplasty, the state of the cartilage is not relevant to the indication.

Evidence base

The search prompt described in appendix 1 produced 1896 results, of which 90 studies reported on the functional outcome of total wrist arthroplasty [22,23,24,25,26,27,28,29,30,31,32,33,34,35,36,37,38,39,40,41,42,43,44,45,46,47,48,49,50,51,52,53,54,55,56,57,58,59,60,61,62,63,64,65,66,67,68,69,70,71,72,73,74,75,76,77,78,79,80,81,82,83,84,85,86,87,88,89,90,91,92,93,94,95,96,97,98,99,100,101,102,103,104,105,106,107,108,109,110,111]. The earliest study was conducted in 1979 [110]. The 1980s and 1990s mainly comprised research on silicone arthroplasty. Recently, research has been focusing on fourth-generation implants. Multiple systematic reviews have been conducted [22,30,31,42].

Technique

**Figure 1:**
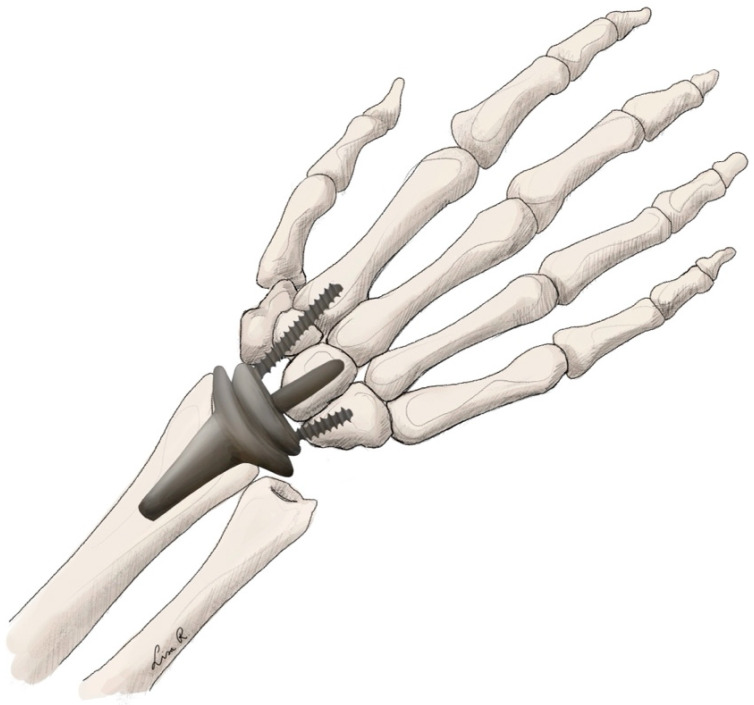
Total wrist arthroplasty with ellipsoid implant.

**Figure 2:**
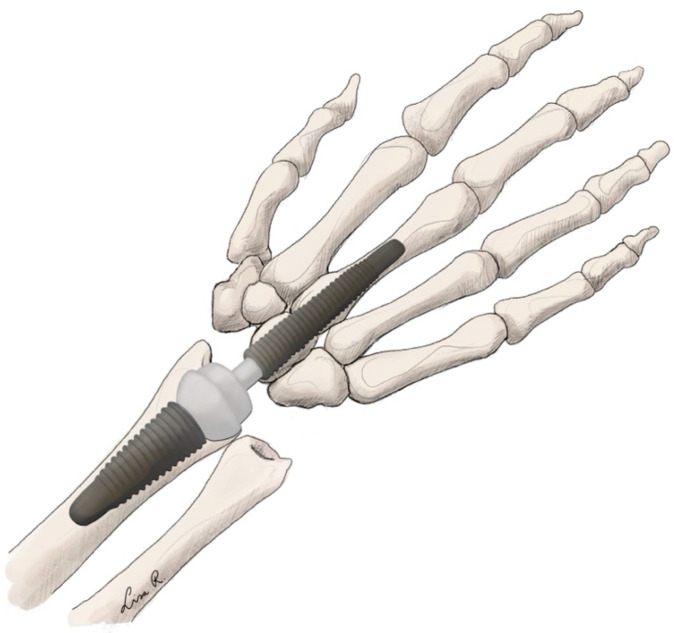
Total wrist arthroplasty with ball-and-socket implant.

Multiple implants are currently on the market for TWA. However, they generally follow the same concepts. For the fourth generation of implants, two concepts are the most obvious. Proximal row carpectomy is always necessary to create space for the implant. One concept is the ellipsoid-shaped implant trying to mimic the original anatomical function of the joint more closely [29,32]. Some examples of this concept include the ReMotion® (Stryker Corporation, Kalamazoo, MI, USA) or the Freedom ® (Smith & Nephew, London, UK) implants in which the carpal plate is fixed to multiple carpal bones on the distal row (see Figure 1) [29,32]. Another possible concept is a ball-and-socket design found in the Motec® (Swemac Innovation AB, Linköping, Sweden) implant. This concept relies on a single screw through the capitate and into the third metacarpal fixing the carpal component, which is connected to the radial component through a ball-and-socket linkage (see Figure 2) [33]. Depending on the different implants, materials usually include titanium, alloys, and polyethylene.

Clinical findings

Currently, the fourth-generation implants are the most recent renditions used in TWA. Significant improvement over their predecessors has been shown in several studies [18,36,48,63]. The alternative treatment for wrist arthritis is arthrodesis of the wrist, eliminating motion in the wrist joint. Various studies have compared TWA to arthrodesis of the wrist joint. Most found no difference in general functional outcome between the two [23,28,35,48,54,59]. However, in a systematic review from 2018, wrist arthrodesis patients did seem to have a greater difficulty with specific tasks like personal hygiene [48]. Nevertheless, TWA was associated with high complication and reoperation rates [24,26,27,28,48,51,73]. The most common complications of TWA are loosening of the carpal component, metacarpal cutout, and instability [19]. Still, the clinical impact of loosening on the patient satisfaction was not apparent [43]. TWA is usually performed on young and demanding populations, which may influence the stress on the implant and thereby its longevity.

#### 3.1.2. Trapeziometacarpal Arthroplasty

The trapeziometacarpal joint is one of the most affected joints in carpal osteoarthritis [4,6]. For the treatment of trapeziometacarpal arthritis, resection arthroplasty, most often a trapeziectomy, is considered standard in most practices around the world. This could be combined with several types of ligamentous reconstructions to fill the remaining cavity and, in some cases, suspend the first metacarpal. Arthroplasty of the trapeziometacarpal joint was introduced in 1972 by Swanson [112], originally using a silicone implant.

Indication

The most common indication for trapeziometacarpal arthroplasty is osteoarthritis of the first carpometacarpal joint [16]. The typical patients are post-menopausal women who have been working with their hands their whole life. However, indications differ between countries. In the Netherlands, for example, arthroplasty of the trapeziometacarpal joint is only recommended in the experimental setting [12], although a shift towards arthroplasty with an implant as a standard treatment has been seen in the last years.

Evidence base

In our literature search, we identified 777 results of which 75 studies met the inclusion criteria [113,114,115,116,117,118,119,120,121,122,123,124,125,126,127,128,129,130,131,132,133,134,135,136,137,138,139,140,141,142,143,144,145,146,147,148,149,150,151,152,153,154,155,156,157,158,159,160,161,162,163,164,165,166,167,168,169,170,171,172,173,174,175,176,177,178,179,180,181,182,183,184,185,186]. Ball-and-socket implants for the trapeziometacarpal joint have been around since the 1970s. Since then, lots of case series have been published. Research into these implants has spiked since the last decade, with multiple randomized controlled trials (RCTs) and systematic reviews being published.

Technique

**Figure 3:**
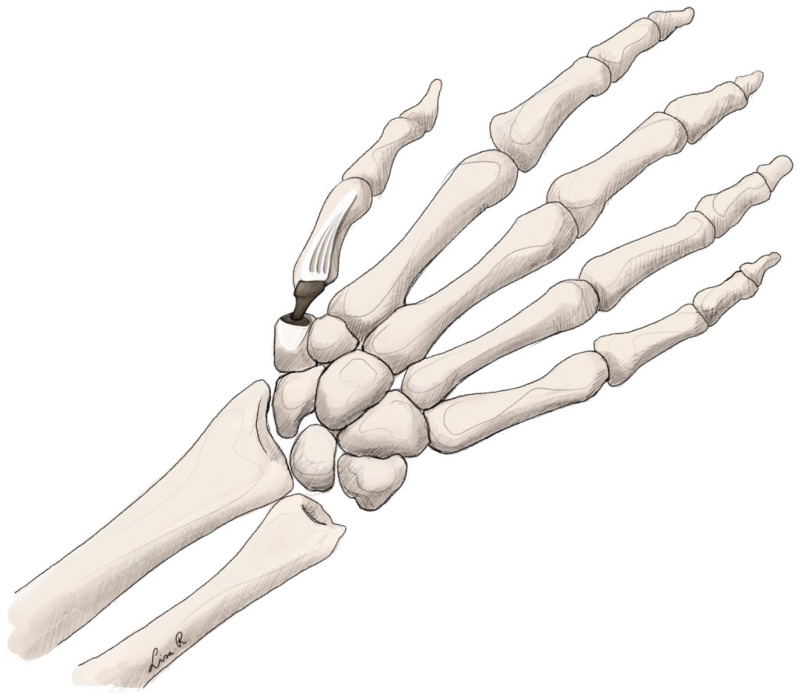
Trapeziometacarpal arthroplasty using a ball-and-socket implant.

Originally the anatomical saddle-like shape of the trapeziometacarpal joint was recreated in the early implants. However, due to complications, this was replaced by a ball-and-socket design [187,188]. These implants consist of a socket, which is placed in the trapezium, and an intramedullary component of the ball to attach it into the first metacarpal. Recently, the concept of dual mobility has been introduced [116]. This consists of a separate socket that can move to some degree inside the outer socket. In theory this may lead to lower complication rates, especially dislocation [124]. The most recent rendition is the TOUCH® (KeriMedical, Montpellier, France) implant [114,136,139,189] (see Figure 3). These implants are made from polyethylene and stainless steel, with full-titanium options available [190].

Clinical Findings

Arthroplasty versus trapeziectomy still remains a debatable subject. Larger studies are starting to be published comparing these treatments. In some studies, arthroplasty seemed to be superior regarding strength and range of motion and other functional outcomes [115,117,122,127,129,137,144,153,157,171]. A recent systematic review has shown similar results with short-term benefits in recovery time associated with trapeziectomy [191]. When compared with arthrodesis, pinch precision seems to be better in joint replacement, while pinch strength is higher in arthrodesis [140]. Complication rates comparing trapeziectomy to arthroplasty are debatable. No significant differences were found in several studies [129,142,144,171,192]. However, other studies did report higher complication rates in joint replacement [137,145,146,147,153,161,162,167,174]. The most common complications seem to be tendinopathy, dislocation, implant loosening, and infection [135,193].

### 3.2. Joint Resurfacing Implants

#### 3.2.1. Amandys® Interposition Spacer

In cases of severe wrist destruction due to degenerative conditions, treatment options are often limited. The most applied procedures are total wrist arthroplasty and total wrist arthrodesis [194,195]. These procedures are relatively intensive and rely on fixation to the bone. The Amandys® (Tornier SAS-Bioprofile Grenoble, Montbonnot-Saint-Martin, France) implant was introduced as a novel pyrocarbon interposition implant in 2012 [194].

Indication

The Amandys® implant is implanted in the radiocarpal and midcarpal space, making it suitable for the treatment of arthritis in the proximal area of the wrist joint like radiocarpal or midcarpal arthritis. To lower the risk of luxation, there should be no malalignment of the wrist, and the joint capsule and interosseus ligaments should ideally be completely intact [194]. However, despite the known injury of soft tissue in patients with rheumatoid arthritis, the implant has shown good results in the treatment of rheumatoid wrists. Although sometimes capsular reinforcement may be needed [196], the indications do overlap with those of TWA.

Evidence base

The search resulted in 11 studies. After screening, ten studies remained [194,195,196,197,198,199,200,201,202,203]. Since introduction in 2012, mainly case reports have been published, with one comparative study being published recently [197].

Technique

**Figure 4:**
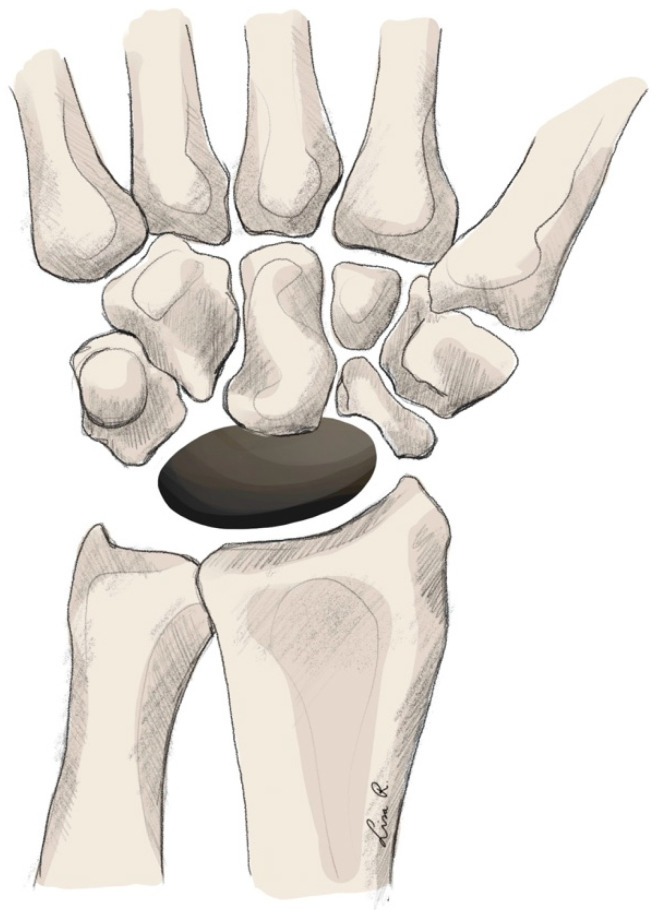
Amandys® implant arthroplasty.

The implant is shaped elliptically in two planes and replaces the lunate and the proximal part of the scaphoid. Contrary to TWA, the implant does not require bony fixation but rather is a free-floating implant in the joint space. This eliminates component loosening as a possible complication [194]. Furthermore, it theoretically provides more-dynamic motion. The implant is manufactured entirely out of pyrocarbon, like most carpal implants currently (see Figure 4).

Clinical findings

The first description of the implant described preliminary results with improved pain reduction and functionality. However, range of motion was not improved significantly [194]. More recent studies have also shown satisfactory results [195,196,197,198,200]. However, a relatively high incidence of revision surgery has also been reported [199]. Some studies note the importance of capsular stability to prevent implant dislocation, which is the most common complication [195,199]. The only comparative study was retrospective and compared the Amandys implant with four-corner arthrodesis. The Amandys® implant was deemed to be non-inferior and associated with shorter immobilization periods [197].

#### 3.2.2. Adaptive Proximal Scaphoid Implant (APSI)

The scaphoid is commonly affected in the case of carpal osteoarthritis. Therefore, multiple implants target this bone. The adaptive proximal scaphoid implant was introduced in France in 2000 [204]. It is a relatively new implant. However, longer-term results are starting to be published currently [205].

Indication

Originally, the implant was specifically designed to treat styloscaphoid arthritis and prevent deterioration of existing nonunion or scapholunate dissociation into advanced collapsed states [204]. Usually, APSI treatment is used before advanced collapse, for example, in cases with nonunion of the scaphoid pole. However, it also provides alternative treatment for wrists already affected by SNAC or SLAC [206]. It is important to ensure that the cartilage of the scaphoid fossa and the capitolunate joint are intact [205].

Evidence base

The literature search yielded 27 results, of which seven studies were included in this overview [205,206,207,208,209,210,211]. These studies all consisted of case series except for one systematic review. No prospective randomized controlled trials were described for this type of arthroplasty.

Technique

**Figure 5:**
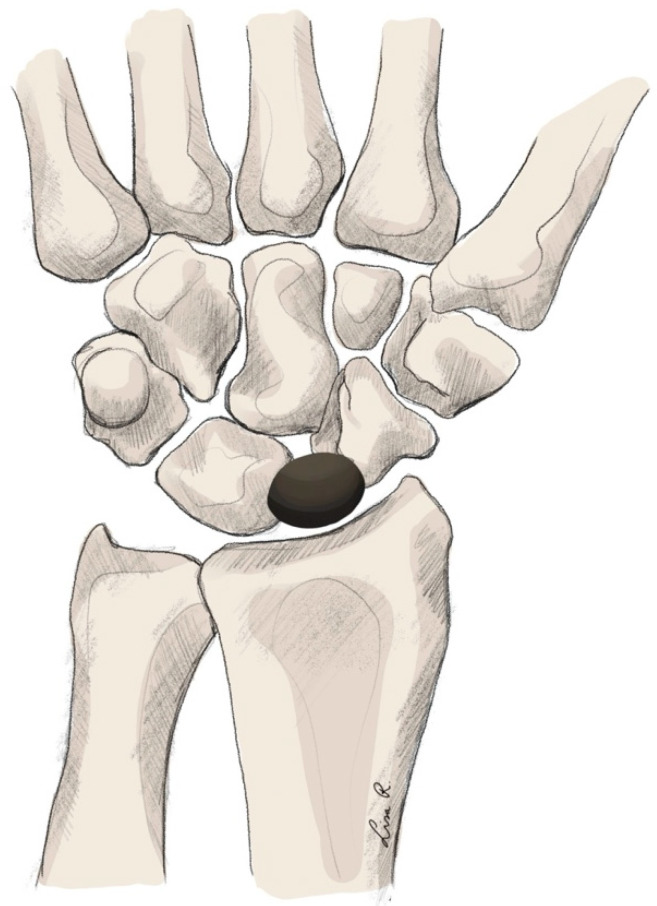
Carpal arthroplasty using the APSI implant.

The implant, like almost all interpositional carpal implants, is produced from pyrocarbon. This ensures good compatibility with the cartilage. The implant, as the name suggests, replaces the proximal pole of the scaphoid and articulates with the scaphoid fossa of the radius [205] (see Figure 5).

Clinical findings

The only systematic review did show APSI to have comparable results to other surgical modalities like four-corner arthrodesis or total wrist arthrodesis [205]. A possible advantage being that it does not disable future salvage procedures. It is however more expensive than traditional arthrodesis [205]. Other studies show good functional results of the APSI arthroplasty [206,207,208,209,210,211]. Complication rates seem to be similar to those for other surgical modalities [205,206,210,211].

#### 3.2.3. Total Carpal Replacement

In the early 1960s, silicone implants for the replacement of the scaphoid were introduced [212]. Biomechanically these implants performed very well. However, the silicone caused inflammation of the synovium, and the particles caused destruction in the wrist. Therefore, the titanium implant was introduced in the late 1980s [212]. Total lunate replacement had a similar development in the treatment of Kienbock’s disease.

Indication

When scaphoid fractures occur, nonunion of the fracture is a notorious complication, since its management can be challenging. When severe arthritis or biomechanical complications like SNAC occur, more drastic surgical treatment is indicated. However, when scaphoid nonunion presents before instability or collapse, total scaphoid arthroplasty may be indicated [15,212].

Total replacement of the lunate can be considered in advanced Kienbock’s disease. However this indication does overlap with the indication for PRC, which is a more commonly performed technique [213].

Evidence base

The literature search provided 278 results, which were screened, after which nine studies provided information on total carpal replacement [14,15,212,213,214,215,216,217,218]. Randomized controlled trials did not exist.

Technique

**Figure 6:**
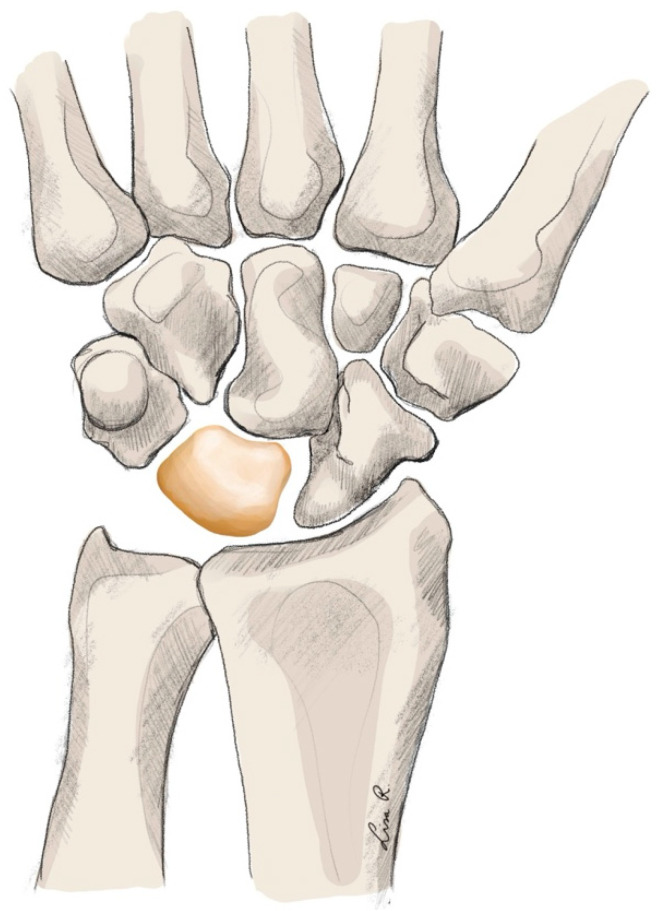
Total carpal replacement arthroplasty.

In both scaphoid and lunate replacement arthroplasty, the entire carpal bone is resected and replaced by an implant. The physical properties of this implant may change. As described, early implants were made of silicone. However, due to complication rates, these are not used currently. The most used material seems to be titanium and pyrocarbon [212,218]. Ready-made implants are available for the lunate (see Figure 6). For the scaphoid, to provide patient-specific implants, 3D-printed titanium implants are used.

Clinical findings

Scaphoid replacement is not very well described in the current literature. Good functional outcomes are reported [212]. More recently, with 3D-printed scaphoids being available, implants have been showing promising functional outcomes [15,214]. No long-term results are available.

Lunate replacement arthroplasty with patient-specific implants is still in development. Ready-made implants do seem to have good results after follow-up [14,215]. The long-term results of these modern techniques remain uncertain. We do know that older techniques like silicone implants yielded unsatisfactory results with high complication rates upwards of 75% [217,218].

#### 3.2.4. Pyrocarbon Capitate Resurfacing Implant (PCRI)

In cases of SLAC or SNAC in the wrist, management can be difficult. Traditionally treatment involved either four-corner arthrodesis or proximal row carpectomy (PRC). In PRC, the entire proximal row of the carpal bones is resected. This may be indicated in high-demand patients with arthritis sparing the lunate fossa, the distal radius, and the proximal pole of the capitate, since these surfaces will form the new wrist joint [219].

Indication

Treatment using PCRI may be indicated when the proximal pole of the capitate is affected. Since a PRC in patients with an affected proximal capitate will create a new joint in which osteoarthritis is already present, resurfacing the proximal capitate may provide better results after PRC [220]. Moreover, treatment with or without resurfacing implants is also based on the surgeon’s preference [221]. These procedures may also be indicated in severe Kienbock’s disease [220,221].

Evidence base

The literature search provided 54 results, of which 10 studies were included [220,221,222,223,224,225,226,227,228,229]. The earliest study was published in 2014 [229]. Studies mainly consist of case series, sometimes including comparison to a retrospective cohort. However, large randomized controlled trials are non-existent.

Technique

**Figure 7:**
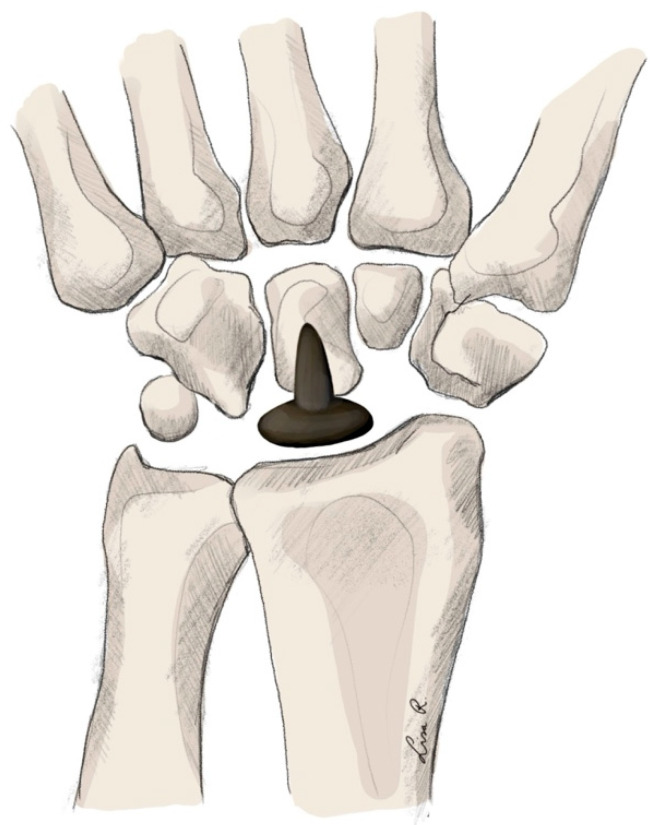
Proximal capitate resurfacing implant arthroplasty.

The PCRI is produced using pyrocarbon. The implant features a monobloc design with a slightly tilted head designed to articulate with the lunar fossa of the distal radius [230]. Firstly, a PRC procedure is performed, in which the scaphoid, lunate, and triquetrum are resected. After this procedure is finished, a minimal osteotomy of the proximal capitate is performed. Following this osteotomy, the stem of the implant is placed in the medullary cavity, and the joint capsule is closed [220] (see Figure 7).

Clinical findings

Treatment with pyrocarbon resurfacing implants is only performed in a select group of patients. Recently, a systematic review was conducted. Proximal row carpectomy without capitate resurfacing implants showed a similar range of motion. However, the use of a PCRI is associated with better functional outcomes [222]. Other studies show similar favorable outcomes of PCRI [221,223,225,227]. Complication rates do seem higher compared with those for proximal row carpectomy [221,224,226]. Luxation of the implant may be caused by difficulty in implant fixation. One review did report a lower percentage of conversion to total wrist arthrodesis compared with capsular interposition. However, follow-up was longer in the interposition groups [226].

#### 3.2.5. Pyrocardan® Disc Interposition Implant

Interposition arthroplasty is prevalent in carpal surgery. The Pyrocardan® disc is widely used to treat pisitriquetral, scaphotrapeziotrapeziodal, and trapeziometacarpal arthritis.

Indication

As stated, indications for the Pyrocardan® disc vary from pisotriquetral arthritis [231] to scaphotrapeziotrapeziodal arthritis [232] and trapiezometacarpal arthritis [233,234]. These implants may also be used for salvage procedures after a failed trapeziectomy [235].

Evidence base

The literature search yielded 15 results. After screening, 13 of these studies provided information on implant performance [231,232,233,234,236,237,238,239,240,241,242,243,244]. Two comparative studies were conducted [237,238].

Technique

**Figure 8:**
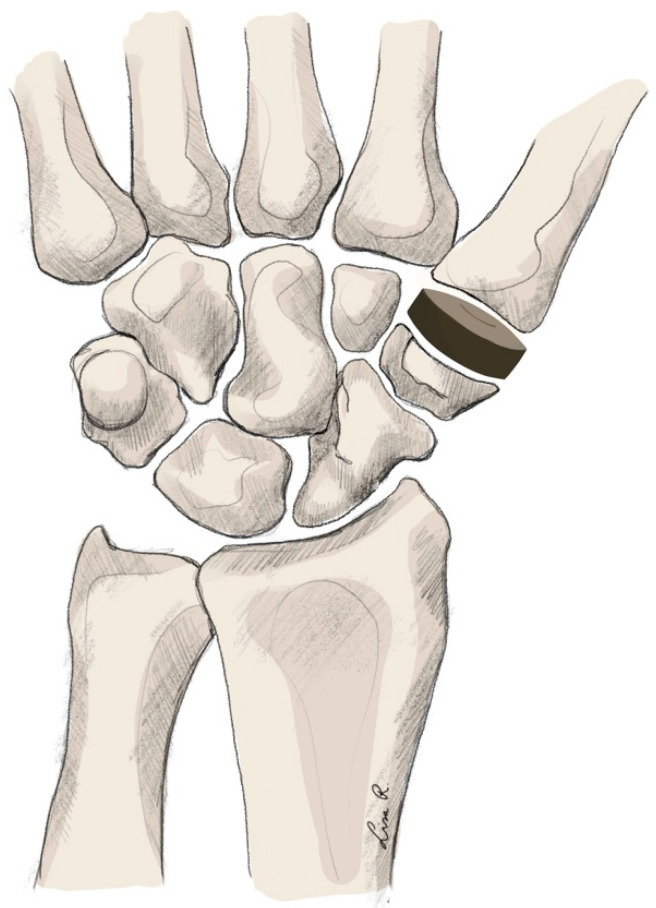
Trapeziometacarpal arthroplasty using a Pyrocardan® implant.

The surgical technique relies on the resurfacing of the carpal joint surface. The implant is made from pyrocarbon. The shaping is a biconcave disc. Based on the indication, carpal resection is performed, after which the implant is placed in this cavity. The implant allows for ligament-sparing insertion, which improves the likelihood of a biomechanically stable joint complex [241] (see Figure 8).

Clinical findings

Pyrocardan® implants in the trapeziometacarpal joint are proven to be effective. Studies suggest a survival rate of 94.3–97.5% [233,236,240,242]. The follow-up in these studies ranged from 2 to 10 years. Functionally, range of motion and grip strength were improved [241,242]. In salvage procedures after trapeziectomy was insufficient, the use of Pyrocardan® implants was shown to improve Quick-DASH scores [235]. When compared with resection arthroplasty, one study reported no significant difference in implant performance [238]. However, another study reported a better outcome in arthroplasty [237]. Complication rates were acceptable [234,240,241]. The most notorious complication with this implant seems to be implant dislocation: one study reported a dislocation rate of 10.7% [234], although some studies have not encountered dislocation of the implants [240,241]. Regarding the effect in pisotriquetral arthritis and scaphotrapeziotrapeziodal arthritis, a single case series for each indication showed good short-term results; however, long-term evidence is missing [231,232].

**Overview**

To acquire an overview of the most relevant information discussed in this review, see Table 1.

## 4. Discussion

Carpal arthroplasty concerns a heterogenic field providing the hand surgeon with a large repertoire of implants. This provides surgeons with the opportunity to offer tailored treatment modalities for each individual patient. Multiple factors play a role in deciding the best implant. The specific indication seems to be the most important, especially in joint resurfacing arthroplasties relying on the cartilage on the other joint surface to still be intact [205]. Moreover, supporting structures like the joint capsule or ligamentous structures need to be intact to prevent luxation of unconstrained implants [194,205,220]. Hence, early intervention is necessary; otherwise, the ligaments or adjacent cartilage may already be destroyed [195,220]. In the case of total arthroplasties like TWA or arthroplasty of the first carpometacarpal joint, the state of the cartilage is less important, since they are not interposition implants and rely on bony fixation for stability.

It is important to note that the use of carpal implants is a safe technique that is already well established like the TWA or the PCRI [18,222], whilst also being relatively new, for example, the Amandys® and APSI arthroplasties. Since much heterogeneity exists in the configuration of osteoarthritis, and tailored treatment is becoming more relevant, it is beneficial for hand surgeons to have a broad knowledge of the available implants to enhance clinical decision-making.

This review covers a lot of different modalities in carpal arthroplasty. Since this is not a systematic review containing a meta-analysis, we cannot provide an objective and significant result concerning the performance of implants in the treatment of carpal osteoarthritis. However, when considering the results of the studies included in this review, a few key points do stand out.

Firstly, on average, implant arthroplasty does not seem to provide much better results than standard surgical treatment. Secondly, descriptions of complication rates vary from favoring non-implant arthroplasty to being similar for both modalities. Almost no studies describe a lower complication rate for implant arthroplasty. This suggests implant arthroplasty may be associated with a higher risk of complications. Considering these two points, surgeons should carefully consider the benefit of implant arthroplasty and take into account that it is often more expensive than non-implant arthroplasty.

The main drawback in the current literature for the lesser-described arthroplasties is the absence of large-scale, adequately powered, randomized controlled trials comparing arthroplasty to the current standard treatments for carpal arthritis.

This study has various limitations. Firstly, no statistical evaluation of the existing literature could be conducted due to the non-systematic nature of the review. Therefore, the aim of this paper is to provide broad information in all implants and on the state of the literature. Secondly, only one search engine was used. Therefore, some potential papers may have been excluded. Thirdly, for some implants, the evidence base was relatively thin. Therefore, it is difficult to adequately assess the performance of these implants based on the literature.

Overall, a recommendation in the use of carpal implants is the need for careful patient selection. The carpal region is biomechanically complex and is prone to implant complications like luxation. The success rate of arthroplasties, especially joint resurfacing arthroplasties like Amandys®, APSI, or PCRI, is largely dependent on the patency and stability of the surrounding tissues like the joint capsule and ligamentous structures. The surgeon needs to pay specific attention to these areas before indicating an arthroplasty.

In the current age of rapid technological development, expectations are that carpal implants will be further engineered and provide better outcomes. We have already seen the first 3D-printed implants [15,214]. The future will most likely be in personalized implants. Currently, techniques are being developed to provide possibilities in non-resecting, interpositional, patient-specific implants. These may be 3D-printed. However, other techniques are also available. This evolution in implant manufacturing may offer an opportunity to further personalize the implant selection. Naturally, since these modalities are currently under development, follow-up data will be necessary to provide data on the clinical outcome.

## 5. Conclusions

Selected patients can benefit from carpal arthroplasty. However, it is not yet the gold standard treatment for most arthritic conditions of the wrist. Complications are overall described as being either more common in implant arthroplasty or similar to those in standard treatment.

## Figures and Tables

**Table 1:** Overview of carpal implants discussed in this review.

Implant	Joint(s)	Concept	Material	Evidence
Total wrist arthroplasty	Radiocarpal	Joint replacement, either ellipsoid or ball and socket	Titanium, alloys, polyethylene	90 studies. No large differences in performance or complications.
Trapeziometacarpal arthroplasty	Trapeziometacarpal	Joint replacement, ball and socket	Polyethylene and stainless steel. Titanium available	75 studies. No large differences in performance. Complication may be higher in implant arthroplasty.
Amandys® implant	Interpositional radiocarpal	Interposition spacer	Pyrocarbon	10 studies. Non-inferior to standard treatment.
Adaptive proximal scaphoid implant (APSI)	Radioscaphoid, scapholunate	Joint resurfacing	Pyrocarbon	7 studies. Comparable to other surgical modalities in performance and complications.
Total carpal replacement	Joint surfaces of the scaphoid or lunate	Total carpal replacement	Titanium, pyrocarbon	9 studies, no RCTs, not enough literature to draw conclusions.
Pyrocarbon capitate resurfacing implant (PRCI)	Radiocapitate (after PRC)	Joint resurfacing	Pyrocarbon	10 studies. Performance seems superior to PRC without resurfacing. Complications are higher in PRCI.
Pyrocardan® disc	Pisitriquetral, scaphotrapeziotrapeziodal, trapeziometacarpal	Interposition disc	Pyrocarbon	13 studies. Implants may perform slightly better than standard treatment in trapeziometacarpal arthritis. Complications are similar.

## Data Availability

The original contributions presented in this study are included in the article. Further inquiries can be directed to the corresponding author.

## Supplementary Materials

The following supporting information can be downloaded at https://www.mdpi.com/article/10.3390/jpm15120575/s1: File S1: PubMed search strategies for each implant type.
