# Supplementary material for: Carpal Implants in the Current Surgical Landscape: An Illustrated Overview
Source: J Pers Med. 2025 Nov 28;15(12):575. doi: 10.3390/jpm15120575 (PMC12734107; doi:10.3390/jpm15120575)
Supplement: Supplementary file 1 [file jpm-15-00575-s001.zip › jpm-3881670-supplementary-file-S1.pdf]

Total wrist arthroplasty

(("total"[All Fields] OR "totaled"[All Fields] OR "totaling"[All Fields] OR "totalled"[All Fields] OR "totalling"[All Fields] OR "totals"[All Fields]) AND ("wrist"[MeSH Terms] OR "wrist"[All Fields] OR "wrist joint"[MeSH Terms] OR ("wrist"[All Fields] AND "joint"[All Fields]) OR "wrist joint"[All Fields] OR "wrists"[All Fields] OR "wrist s"[All Fields]) AND ("arthroplasty"[MeSH Terms] OR "arthroplasty"[All Fields] OR "arthroplasties"[All Fields])) OR (("wrist"[MeSH Terms] OR "wrist"[All Fields] OR "wrist joint"[MeSH Terms] OR ("wrist"[All Fields] AND "joint"[All Fields]) OR "wrist joint"[All Fields] OR "wrists"[All Fields] OR "wrist s"[All Fields]) AND ("arthroplasty"[MeSH Terms] OR "arthroplasty"[All Fields] OR "arthroplasties"[All Fields])) OR (("total"[All Fields] OR "totaled"[All Fields] OR "totaling"[All Fields] OR "totalled"[All Fields] OR "totalling"[All Fields] OR "totals"[All Fields]) AND "wirst"[All Fields] AND ("drug implants"[Supplementary Concept] OR "drug implants"[All Fields] OR "implant"[All Fields] OR "drug implants"[MeSH Terms] OR ("drug"[All Fields] AND "implants"[All Fields]) OR "embryo implantation"[MeSH Terms] OR ("embryo"[All Fields] AND "implantation"[All Fields]) OR "embryo implantation"[All Fields] OR "implantation"[All Fields] OR "implant s"[All Fields] OR "implantability"[All Fields] OR "implantable"[All Fields] OR "implantables"[All Fields] OR "implantate"[All Fields] OR "implantated"[All Fields] OR "implantates"[All Fields] OR "implantations"[All Fields] OR "implanted"[All Fields] OR "implanter"[All Fields] OR "implanters"[All Fields] OR "implanting"[All Fields] OR "implantion"[All Fields] OR "implantitis"[All Fields] OR "implants"[All Fields])) OR (("total"[All Fields] OR "totaled"[All Fields] OR "totaling"[All Fields] OR "totalled"[All Fields] OR "totalling"[All Fields] OR "totals"[All Fields]) AND ("wrist"[MeSH Terms] OR "wrist"[All Fields] OR "wrist joint"[MeSH Terms] OR ("wrist"[All Fields] AND "joint"[All Fields]) OR "wrist joint"[All Fields] OR "wrists"[All Fields] OR "wrist s"[All Fields]) AND ("drug implants"[Supplementary Concept] OR "drug implants"[All Fields] OR "implant"[All Fields] OR "drug implants"[MeSH Terms] OR ("drug"[All Fields] AND "implants"[All Fields]) OR "embryo implantation"[MeSH Terms] OR ("embryo"[All Fields] AND "implantation"[All Fields]) OR "embryo implantation"[All Fields] OR "implantation"[All Fields] OR "implant s"[All Fields] OR "implantability"[All Fields] OR "implantable"[All Fields] OR "implantables"[All Fields] OR "implantate"[All Fields] OR "implantated"[All Fields] OR "implantates"[All Fields] OR "implantations"[All Fields] OR "implanted"[All Fields] OR "implanter"[All Fields] OR "implanters"[All Fields] OR "implanting"[All Fields] OR "implantion"[All Fields] OR "implantitis"[All Fields] OR "implants"[All Fields])) OR (("total"[All Fields] OR "totaled"[All Fields] OR "totaling"[All Fields] OR "totalled"[All Fields] OR "totalling"[All Fields] OR "totals"[All Fields]) AND ("wrist"[MeSH Terms] OR "wrist"[All Fields] OR "wrist joint"[MeSH Terms] OR ("wrist"[All Fields] AND "joint"[All Fields]) OR "wrist joint"[All Fields] OR "wrists"[All Fields] OR "wrist s"[All Fields]) AND ("replace"[All Fields] OR "replaceable"[All Fields] OR "replaced"[All Fields] OR "replaces"[All Fields] OR "replacing"[All Fields] OR "replacment"[All Fields] OR "replantation"[MeSH Terms] OR "replantation"[All Fields] OR "replacement"[All Fields] OR "replacements"[All Fields]))

First carpometacarpal arthroplasty

("Trapeziometacarpal"[All Fields] AND ("arthroplasty"[MeSH Terms] OR "arthroplasty"[All Fields] OR "arthroplasties"[All Fields])) OR ("CMC1"[All Fields] AND ("prostheses and implants"[MeSH Terms] OR ("prostheses"[All Fields] AND "implants"[All Fields]) OR "prostheses and implants"[All Fields] OR "prosthesis"[All Fields])) OR ("CMC1"[All Fields] AND ("drug implants"[Supplementary Concept] OR "drug implants"[All Fields] OR "implant"[All Fields] OR "drug implants"[MeSH Terms] OR ("drug"[All Fields] AND "implants"[All Fields]) OR "embryo implantation"[MeSH Terms] OR ("embryo"[All Fields] AND "implantation"[All Fields]) OR "embryo implantation"[All Fields] OR "implantation"[All Fields] OR "implant s"[All Fields] OR "implantability"[All Fields] OR "implantable"[All Fields] OR "implantables"[All Fields] OR "implantate"[All Fields] OR "implantated"[All Fields] OR "implantates"[All Fields] OR "implantations"[All Fields] OR "implanted"[All Fields] OR "implanter"[All Fields] OR "implanters"[All Fields] OR "implanting"[All Fields] OR "implantion"[All Fields] OR "implantitis"[All Fields] OR "implants"[All Fields])) OR

("CMC1"[All Fields] AND ("arthroplasty"[MeSH Terms] OR "arthroplasty"[All Fields] OR "arthroplasties"[All Fields])) OR (("first"[All Fields] OR "firsts"[All Fields]) AND "carpometacarpal"[All Fields] AND ("drug implants"[Supplementary Concept] OR "drug implants"[All Fields] OR "implant"[All Fields] OR "drug implants"[MeSH Terms] OR ("drug"[All Fields] AND "implants"[All Fields]) OR "embryo implantation"[MeSH Terms] OR ("embryo"[All Fields] AND "implantation"[All Fields]) OR "embryo implantation"[All Fields] OR "implantation"[All Fields] OR "implant s"[All Fields] OR "implantability"[All Fields] OR "implantable"[All Fields] OR "implantables"[All Fields] OR "implantate"[All Fields] OR "implantated"[All Fields] OR "implantates"[All Fields] OR "implantations"[All Fields] OR "implanted"[All Fields] OR "implanter"[All Fields] OR "implanters"[All Fields] OR "implanting"[All Fields] OR "implantion"[All Fields] OR "implantitis"[All Fields] OR "implants"[All Fields])) OR (("first"[All Fields] OR "firsts"[All Fields]) AND "carpometacarpal"[All Fields] AND ("arthroplasty"[MeSH Terms] OR "arthroplasty"[All Fields] OR "arthroplasties"[All Fields])) OR (("first"[All Fields] OR "firsts"[All Fields]) AND "carpometacarpal"[All Fields] AND ("prostheses and implants"[MeSH Terms] OR ("prostheses"[All Fields] AND "implants"[All Fields]) OR "prostheses and implants"[All Fields] OR "prosthesis"[All Fields]))

<u>Amandys® implant</u>

("amandys"[All Fields] AND ("drug implants"[Supplementary Concept] OR "drug implants"[All Fields] OR "implant"[All Fields] OR "drug implants"[MeSH Terms] OR ("drug"[All Fields] AND "implants"[All Fields]) OR "embryo implantation"[MeSH Terms] OR ("embryo"[All Fields] AND "implantation"[All Fields]) OR "embryo implantation"[All Fields] OR "implantation"[All Fields] OR "implant s"[All Fields] OR "implantability"[All Fields] OR "implantable"[All Fields] OR "implantables"[All Fields] OR "implantate"[All Fields] OR "implantated"[All Fields] OR "implantates"[All Fields] OR "implantations"[All Fields] OR "implanted"[All Fields] OR "implanter"[All Fields] OR "implanters"[All Fields] OR "implanting"[All Fields] OR "implantion"[All Fields] OR "implantitis"[All Fields] OR "implants"[All Fields])) OR "amandys"[All Fields] OR ("amandys"[All Fields] AND ("prostheses and implants"[MeSH Terms] OR ("prostheses"[All Fields] AND "implants"[All Fields]) OR "prostheses and implants"[All Fields] OR "prosthesis"[All Fields])) OR ("amandys"[All Fields] AND ("arthroplasty"[MeSH Terms] OR "arthroplasty"[All Fields] OR "arthroplasties"[All Fields]))

<u>Pyrocardan® disc</u>

("pyrocardan"[All Fields] AND "disc"[All Fields]) OR ("pyrocardan"[All Fields] AND ("drug implants"[Supplementary Concept] OR "drug implants"[All Fields] OR "implant"[All Fields] OR "drug implants"[MeSH Terms] OR ("drug"[All Fields] AND "implants"[All Fields]) OR "embryo implantation"[MeSH Terms] OR ("embryo"[All Fields] AND "implantation"[All Fields]) OR "embryo implantation"[All Fields] OR "implantation"[All Fields] OR "implant s"[All Fields] OR "implantability"[All Fields] OR "implantable"[All Fields] OR "implantables"[All Fields] OR "implantate"[All Fields] OR "implantated"[All Fields] OR "implantates"[All Fields] OR "implantations"[All Fields] OR "implanted"[All Fields] OR "implanter"[All Fields] OR "implanters"[All Fields] OR "implanting"[All Fields] OR "implantion"[All Fields] OR "implantitis"[All Fields] OR "implants"[All Fields])) OR ("pyrocardan"[All Fields] AND ("wrist"[MeSH Terms] OR "wrist"[All Fields] OR "wrist joint"[MeSH Terms] OR ("wrist"[All Fields] AND "joint"[All Fields]) OR "wrist joint"[All Fields] OR "wrists"[All Fields] OR "wrist s"[All Fields]) AND ("drug implants"[Supplementary Concept] OR "drug implants"[All Fields] OR "implant"[All Fields] OR "drug implants"[MeSH Terms] OR ("drug"[All Fields] AND "implants"[All Fields]) OR "embryo implantation"[MeSH Terms] OR ("embryo"[All Fields] AND "implantation"[All Fields]) OR "embryo implantation"[All Fields] OR "implantation"[All Fields] OR "implant s"[All Fields] OR "implantability"[All Fields] OR "implantable"[All Fields] OR "implantables"[All Fields] OR "implantate"[All Fields] OR "implantated"[All Fields] OR "implantates"[All Fields] OR "implantations"[All Fields] OR "implanted"[All Fields] OR "implanter"[All Fields] OR "implanters"[All Fields] OR "implanting"[All Fields] OR "implantion"[All Fields] OR "implantitis"[All

Fields] OR "implants"[All Fields])) OR ("pyrocardan"[All Fields] AND ("prostheses and implants"[MeSH Terms] OR ("prostheses"[All Fields] AND "implants"[All Fields]) OR "prostheses and implants"[All Fields] OR "prosthesis"[All Fields])) OR ("pyrocardan"[All Fields] AND ("arthroplasty"[MeSH Terms] OR "arthroplasty"[All Fields] OR "arthroplasties"[All Fields]))

Pyrocarbon capitate resurfacing implant

(("pyrolytic carbon"[Supplementary Concept] OR "pyrolytic carbon"[All Fields] OR "pyrocarbon"[All Fields]) AND ("capitate bone"[MeSH Terms] OR ("capitate"[All Fields] AND "bone"[All Fields]) OR "capitate bone"[All Fields] OR "capitate"[All Fields] OR "capitates"[All Fields]) AND ("resurface"[All Fields] OR "resurfaced"[All Fields] OR "resurfaces"[All Fields] OR "resurfacing"[All Fields] OR "resurfacings"[All Fields]) AND ("drug implants"[Supplementary Concept] OR "drug implants"[All Fields] OR "implant"[All Fields] OR "drug implants"[MeSH Terms] OR ("drug"[All Fields] AND "implants"[All Fields]) OR "embryo implantation"[MeSH Terms] OR ("embryo"[All Fields] AND "implantation"[All Fields]) OR "embryo implantation"[All Fields] OR "implantation"[All Fields] OR "implant s"[All Fields] OR "implantability"[All Fields] OR "implantable"[All Fields] OR "implantables"[All Fields] OR "implantate"[All Fields] OR "implantated"[All Fields] OR "implantates"[All Fields] OR "implantations"[All Fields] OR "implanted"[All Fields] OR "implanter"[All Fields] OR "implanters"[All Fields] OR "implanting"[All Fields] OR "implantion"[All Fields] OR "implantitis"[All Fields] OR "implants"[All Fields])) OR (("capitate bone"[MeSH Terms] OR ("capitate"[All Fields] AND "bone"[All Fields]) OR "capitate bone"[All Fields] OR "capitate"[All Fields] OR "capitates"[All Fields]) AND ("resurface"[All Fields] OR "resurfaced"[All Fields] OR "resurfaces"[All Fields] OR "resurfacing"[All Fields] OR "resurfacings"[All Fields])) OR (("capitate bone"[MeSH Terms] OR ("capitate"[All Fields] AND "bone"[All Fields]) OR "capitate bone"[All Fields] OR "capitate"[All Fields] OR "capitates"[All Fields]) AND ("resurface"[All Fields] OR "resurfaced"[All Fields] OR "resurfaces"[All Fields] OR "resurfacing"[All Fields] OR "resurfacings"[All Fields]) AND ("arthroplasty"[MeSH Terms] OR "arthroplasty"[All Fields] OR "arthroplasties"[All Fields])) OR (("capitate bone"[MeSH Terms] OR ("capitate"[All Fields] AND "bone"[All Fields]) OR "capitate bone"[All Fields] OR "capitate"[All Fields] OR "capitates"[All Fields]) AND ("resurface"[All Fields] OR "resurfaced"[All Fields] OR "resurfaces"[All Fields] OR "resurfacing"[All Fields] OR "resurfacings"[All Fields]) AND ("prostheses and implants"[MeSH Terms] OR ("prostheses"[All Fields] AND "implants"[All Fields]) OR "prostheses and implants"[All Fields] OR "prosthesis"[All Fields])) OR (("capitate bone"[MeSH Terms] OR ("capitate"[All Fields] AND "bone"[All Fields]) OR "capitate bone"[All Fields] OR "capitate"[All Fields] OR "capitates"[All Fields]) AND ("resurface"[All Fields] OR "resurfaced"[All Fields] OR "resurfaces"[All Fields] OR "resurfacing"[All Fields] OR "resurfacings"[All Fields]) AND ("drug implants"[Supplementary Concept] OR "drug implants"[All Fields] OR "implant"[All Fields] OR "drug implants"[MeSH Terms] OR ("drug"[All Fields] AND "implants"[All Fields]) OR "embryo implantation"[MeSH Terms] OR ("embryo"[All Fields] AND "implantation"[All Fields]) OR "embryo implantation"[All Fields] OR "implantation"[All Fields] OR "implant s"[All Fields] OR "implantability"[All Fields] OR "implantable"[All Fields] OR "implantables"[All Fields] OR "implantate"[All Fields] OR "implantated"[All Fields] OR "implantates"[All Fields] OR "implantations"[All Fields] OR "implanted"[All Fields] OR "implanter"[All Fields] OR "implanters"[All Fields] OR "implanting"[All Fields] OR "implantion"[All Fields] OR "implantitis"[All Fields] OR "implants"[All Fields])) OR (("pyrolytic carbon"[Supplementary Concept] OR "pyrolytic carbon"[All Fields] OR "pyrocarbon"[All Fields]) AND ("resurface"[All Fields] OR "resurfaced"[All Fields] OR "resurfaces"[All Fields] OR "resurfacing"[All Fields] OR "resurfacings"[All Fields]) AND ("drug implants"[Supplementary Concept] OR "drug implants"[All Fields] OR "implant"[All Fields] OR "drug implants"[MeSH Terms] OR ("drug"[All Fields] AND "implants"[All Fields]) OR "embryo implantation"[MeSH Terms] OR ("embryo"[All Fields] AND "implantation"[All Fields]) OR "embryo implantation"[All Fields] OR "implantation"[All Fields] OR "implant s"[All Fields] OR "implantability"[All Fields] OR "implantable"[All Fields] OR "implantables"[All Fields] OR "implantate"[All Fields] OR "implantated"[All Fields] OR "implantates"[All Fields] OR "implantations"[All Fields] OR

"implanted"[All Fields] OR "implanter"[All Fields] OR "implanters"[All Fields] OR "implanting"[All Fields] OR "implantion"[All Fields] OR "implantitis"[All Fields] OR "implants"[All Fields]))

Adaptive proximal scaphoid implant (APSI)

("APSI"[All Fields] AND ("drug implants"[Supplementary Concept] OR "drug implants"[All Fields] OR "implant"[All Fields] OR "drug implants"[MeSH Terms] OR ("drug"[All Fields] AND "implants"[All Fields]) OR "embryo implantation"[MeSH Terms] OR ("embryo"[All Fields] AND "implantation"[All Fields]) OR "embryo implantation"[All Fields] OR "implantation"[All Fields] OR "implant s"[All Fields] OR "implantability"[All Fields] OR "implantable"[All Fields] OR "implantables"[All Fields] OR "implantate"[All Fields] OR "implantated"[All Fields] OR "implantates"[All Fields] OR "implantations"[All Fields] OR "implanted"[All Fields] OR "implanter"[All Fields] OR "implanters"[All Fields] OR "implanting"[All Fields] OR "implantion"[All Fields] OR "implantitis"[All Fields] OR "implants"[All Fields])) OR ("APSI"[All Fields] AND ("scaphoid bone"[MeSH Terms] OR ("scaphoid"[All Fields] AND "bone"[All Fields]) OR "scaphoid bone"[All Fields] OR "scaphoid"[All Fields] OR "scaphoid s"[All Fields] OR "scaphoids"[All Fields])) OR ("APSI"[All Fields] AND ("prostheses and implants"[MeSH Terms] OR ("prostheses"[All Fields] AND "implants"[All Fields]) OR "prostheses and implants"[All Fields] OR "prosthesis"[All Fields])) OR ("APSI"[All Fields] AND ("arthroplasty"[MeSH Terms] OR "arthroplasty"[All Fields] OR "arthroplasties"[All Fields])) OR (("acclimatization"[MeSH Terms] OR "acclimatization"[All Fields] OR "adaptation"[All Fields] OR "adaptations"[All Fields] OR "adapt"[All Fields] OR "adaptabilities"[All Fields] OR "adaptability"[All Fields] OR "adaptable"[All Fields] OR "adaptational"[All Fields] OR "adaptative"[All Fields] OR "adapte"[All Fields] OR "adapted"[All Fields] OR "adapting"[All Fields] OR "adaption"[All Fields] OR "adaptions"[All Fields] OR "adaptive"[All Fields] OR "adaptively"[All Fields] OR "adaptiveness"[All Fields] OR "adaptivity"[All Fields] OR "adapts"[All Fields]) AND ("proximal"[All Fields] OR "proximalization"[All Fields] OR "proximalize"[All Fields] OR "proximalized"[All Fields] OR "proximalizes"[All Fields] OR "proximalizing"[All Fields] OR "proximally"[All Fields] OR "proximals"[All Fields]) AND ("scaphoid bone"[MeSH Terms] OR ("scaphoid"[All Fields] AND "bone"[All Fields]) OR "scaphoid bone"[All Fields] OR "scaphoid"[All Fields] OR "scaphoid s"[All Fields] OR "scaphoids"[All Fields]) AND ("drug implants"[Supplementary Concept] OR "drug implants"[All Fields] OR "implant"[All Fields] OR "drug implants"[MeSH Terms] OR ("drug"[All Fields] AND "implants"[All Fields]) OR "embryo implantation"[MeSH Terms] OR ("embryo"[All Fields] AND "implantation"[All Fields]) OR "embryo implantation"[All Fields] OR "implantation"[All Fields] OR "implant s"[All Fields] OR "implantability"[All Fields] OR "implantable"[All Fields] OR "implantables"[All Fields] OR "implantate"[All Fields] OR "implantated"[All Fields] OR "implantates"[All Fields] OR "implantations"[All Fields] OR "implanted"[All Fields] OR "implanter"[All Fields] OR "implanters"[All Fields] OR "implanting"[All Fields] OR "implantion"[All Fields] OR "implantitis"[All Fields] OR "implants"[All Fields]))

Total carpal replacement

"lunate replacement"[All Fields] OR "scaphoid replacement"[All Fields] OR (("hamate bone"[MeSH Terms] OR ("hamate"[All Fields] AND "bone"[All Fields]) OR "hamate bone"[All Fields] OR "hamate"[All Fields] OR "hamates"[All Fields]) AND ("replace"[All Fields] OR "replaceable"[All Fields] OR "replaced"[All Fields] OR "replaces"[All Fields] OR "replacing"[All Fields] OR "replacment"[All Fields] OR "replantation"[MeSH Terms] OR "replantation"[All Fields] OR "replacement"[All Fields] OR "replacements"[All Fields])) OR (("capitate bone"[MeSH Terms] OR ("capitate"[All Fields] AND "bone"[All Fields]) OR "capitate bone"[All Fields] OR "capitate"[All Fields] OR "capitates"[All Fields]) AND ("replace"[All Fields] OR "replaceable"[All Fields] OR "replaced"[All Fields] OR "replaces"[All Fields] OR "replacing"[All Fields] OR "replacment"[All Fields] OR "replantation"[MeSH Terms] OR "replantation"[All Fields] OR "replacement"[All Fields] OR "replacements"[All Fields])) OR (("pisiform bone"[MeSH Terms] OR ("pisiform"[All Fields] AND "bone"[All Fields]) OR "pisiform bone"[All Fields] OR "pisiform"[All Fields] OR "pisiforme"[All Fields]) AND ("replace"[All Fields] OR "replaceable"[All Fields] OR "replaced"[All Fields] OR

"replaces"[All Fields] OR "replacing"[All Fields] OR "replacment"[All Fields] OR "replantation"[MeSH Terms] OR "replantation"[All Fields] OR "replacement"[All Fields] OR "replacements"[All Fields])) OR "trapezium replacement"[All Fields] OR (("trapezoid bone"[MeSH Terms] OR ("trapezoid"[All Fields] AND "bone"[All Fields]) OR "trapezoid bone"[All Fields] OR "trapezoid"[All Fields] OR "trapezoidal"[All Fields] OR "trapezoidally"[All Fields] OR "trapezoids"[All Fields]) AND ("replace"[All Fields] OR "replaceable"[All Fields] OR "replaced"[All Fields] OR "replaces"[All Fields] OR "replacing"[All Fields] OR "replacment"[All Fields] OR "replantation"[MeSH Terms] OR "replantation"[All Fields] OR "replacement"[All Fields] OR "replacements"[All Fields])) OR (("triquetrum bone"[MeSH Terms] OR ("triquetrum"[All Fields] AND "bone"[All Fields]) OR "triquetrum bone"[All Fields] OR "triquetrum"[All Fields]) AND ("replace"[All Fields] OR "replaceable"[All Fields] OR "replaced"[All Fields] OR "replaces"[All Fields] OR "replacing"[All Fields] OR "replacment"[All Fields] OR "replantation"[MeSH Terms] OR "replantation"[All Fields] OR "replacement"[All Fields] OR "replacements"[All Fields]))
